# Shifting from techno-economic to socio-ecological priorities: Incorporating landscape preferences and ecosystem services into the siting of renewable energy infrastructure

**DOI:** 10.1371/journal.pone.0298430

**Published:** 2024-04-10

**Authors:** Boris Salak, Marcel Hunziker, Adrienne Grêt-Regamey, Reto Spielhofer, Ulrike Wissen Hayek, Felix Kienast

**Affiliations:** 1 Swiss Federal Institute for Forest, Snow and Landscape Research WSL, Social Sciences in Landscape Research Group, Research Unit Economics and Social Sciences, Birmensdorf, Switzerland; 2 TU Wien, Faculty of Architecture and Planning, Institute of Urban Design and Landscape Architecture, Research Unit Landscape Architecture and Landscape Planning, Vienna, Austria; 3 Planning of Landscape and Urban Systems (PLUS), Institute for Spatial and Landscape Development, ETH Zürich, Zürich, Switzerland; 4 Norwegian Institute for Nature Research, Trondheim, Norway; 5 Landscape Ecology Group, Department of Environmental Systems Science, ETH Zürich, Zürich, Switzerland; 6 Swiss Federal Institute for Forest, Snow and Landscape Research WSL, Land Change Science Research Group, Research Unit Land-use Systems, Birmensdorf, Switzerland; Vellore Institute of Technology, INDIA

## Abstract

This study examines the siting scenarios for renewable energy installations (REI) in a mountainous region of Europe (Switzerland), incorporating the external costs of ecosystem services and, innovatively, social preferences. This approach challenges the prevalent techno-economic siting paradigm, which often overlooks these externalities. To minimize the external costs of the scenarios while maximizing energy yield, Marxan, an optimization software, was employed. The energy target for all scenarios is set at 25 TWh/a, stemming from the energy gap anticipated due to the phase-out of Swiss nuclear reactors by 2050. This target is met using renewable energy infrastructure such as wind, roof-mounted photovoltaic, and ground-mounted photovoltaic systems. By integrating social preferences into the optimization, this study showcases a promising implementation that transcends the software’s intended applications. It complements techno-economic approaches and offers alternative decision-making avenues. The conventional "roof first" strategy proved ineffective in preventing extensive land use for the development of new renewable energy infrastructure. Strategies incorporating ground-mounted photovoltaic infrastructure were more spatially, ecologically, and socially efficient than those without. The strategy optimized for energy yield exhibited the highest spatial efficiency but incurred significant ecosystem service costs and, surprisingly, had low social costs. In contrast, the strategy prioritizing ecosystem services was the most efficient in terms of ecosystem service costs but had elevated social costs and was spatially less efficient than other strategies. The strategy optimized for social preferences incurred the lowest social costs and excelled in spatial efficiency and ecosystem service costs. Notably, this strategy employed a limited number of planning units linked to both high ecosystem service and social costs. The findings underscore that incorporating social preferences significantly enhances the evaluation of siting options. This inclusion allows for the social acceptance of investments to be factored into costs, facilitating more informed and inclusive decisions.

## Introduction

The global community stands at a crossroads, faced with the undeniable impacts of climate change. Driven by the surge in anthropogenic CO2 concentrations, global warming has emerged as a defining challenge of our times. This warming trend, particularly pronounced in mountainous regions, underscores the urgency of transitioning to sustainable energy solutions [1]. Renewable energy technologies, from solar to wind, present a beacon of hope in this context. They not only offer a means to reduce greenhouse gas emissions but also provide a pathway to adapt to the changing climate patterns, especially in regions with unique vulnerabilities like mountainous terrains [2]. However, the journey to a renewable energy future is not without its complexities. Siting large energy installations, especially in countries with rich natural landscapes and cultural heritage, presents a multifaceted challenge. These regions often grapple with the delicate balance of harnessing renewable energy while preserving their intrinsic environmental and landscape values. Public debates around these installations frequently revolve around concerns related to outdoor recreation, place attachment, and tourism. The concept of optimal site selection for energy projects is not merely a technical decision; it is deeply intertwined with the societal values and priorities [3, 4]. Research can provide powerful decision support systems to facilitate the negotiation process, allowing decision-makers to reach a solution based on assessments of tailored trade-offs between, for example, landscape issues, nature conservation and renewable energies.

Such decision support for energy issues has been presented in the pioneering work of Hastik et al. [5], who developed conflict matrices between renewable energy projects in the Alpine region and from benefits that humans derive from ecosystems (ecosystem services) [6] based on a broad literature review. Kienast et al. [7] and Huber et al. [8] developed the approach further, using systematic ecosystem service mapping to identify areas with little or no environmental conflicts with wind or solar energy production in Switzerland. These authors also included future land-use change and technological advances to calculate future trade-offs. Similar to Kienast et al. [7], Wiehe et al. [9] made a spatially explicit assessment of Germany to identify low-conflict areas for human health and nature.

Egli et al. [10] was the first to optimize energy output vs. environmental costs in a spatially explicit way. Instead of preemptively excluding all energy-rich sites with high environmental costs, they employed an annealing optimization algorithm to simultaneously reduce environmental costs while maximizing benefits, particularly energy output. Similar attempts have been presented by Göcke et al. [11], Lehmann et al. [12], Wang et al. [13], and others.

All the above-mentioned research papers have a common shortcoming: while the environmental costs were reliably estimated, the costs due to acceptance or reactance of the population were not or only vaguely included in informed decision-making. Thanks to a recent research effort by the authors of this present manuscript, these data are now available in high quality and can be included in trade-off modeling which includes–for the first time in energy-related siting procedures–costs for ecosystem services but also representative social perspectives of social acceptance and reactance (social costs). The latter is known to be a key factor for successfully transitioning to a more sustainable energy system [14, 15]. Indeed, including social perspectives is crucial for several players in the planning and policy arena, as doing so could contribute to a more informed basis for energy planning. First, investors might see further conflicting areas which could have been undetected without the information. Second, public acceptance of a project can greatly accelerate its realization process and reduce costs for legal disputes [16]. Third, this aspect is important for political debates, as people’s evaluations can be integrated into participatory landscape planning documents.

If the energy transition ought to be based on a broad social consensus, site selection must be participatory, involving e.g. power companies and landscape and nature conservationists, and must consider economic needs and society preferences. Owing to its participatory tradition of decision-making, Switzerland is an excellent pilot region for developing and testing decision support systems with a strong participatory component. According to the Energy Strategy 2050 [17], Switzerland aims to generate around 25 TWh/a from renewable energies by 2050. This corresponds to the energy gap that will result from the planned phase-out of all nuclear reactors in the country. The plan is to fill this gap with mostly roof-mounted photovoltaic (rm-PV) infrastructure and wind-energy infrastructure. It is argued that the transition is proceeding too slowly and that, despite evident pressure, Switzerland will not reach the goals within the designated time [18]. Hence, ground-mounted photovoltaic systems (gm-PV) might be key for the decarbonization of Swiss society. However, gm-PV infrastructures have been excluded from discussions in Switzerland for a long time because of landscape protection and ethical considerations (competition for food production). This started to change in autumn 2022. Triggered by geopolitical developments, a heavy drought in Switzerland, and an impending electricity supply shortage, political initiatives supporting gm-PV developments have started to appear. The focus has been on Swiss Alpine landscapes, and the initiatives have been rationalized by the need for electricity during winter season [19].

The goal of this paper is to demonstrate that–even amid geopolitical and climate-related pushes to generate as much renewable energy as possible–informed siting decisions can be made. This is possible through spatially explicit decision support tools that consider the triangular relationship between energy effectiveness, ecosystem service costs, and social costs, as follows:

1. Energy effectivity strategy [NRG]: focusing purely on energy effectivity by selecting sites with the highest energy yields (high energy production per year) without considering environmental and social external costs. It is hypothesized that this strategy will result in minimization of the number of sites necessary to achieve the desired energy output, but that this reduction will be accompanied by a notable increase in ecological and social costs.
2. Low ecosystem service cost strategy [ESS]: selecting the best sites in terms of energy output at the lowest environmental costs. It is hypothesized that this strategy will lead to a spatial distribution that disregards regions of substantial importance to human populations.
3. Low social cost strategy [SOC]: selecting the best sites in terms of energy at the lowest social costs. It is hypothesized that this strategy will result in a spatial pattern that ignores the ecological attributes of the landscape.

It is further expected that any strategy considering external costs [ESS, SOC] will be spatially less effective and will rely more on other landscapes and community types compared with the strategy excluding external costs [NRG]. The ability to simulate and predict such trade-offs is a key feature of the optimization method used here (Marxan), which forms the basis for decision support tools. Solutions with a a few concentrated sites with a high energy output might be preferred over solutions with many spatially distributed sites for ecological and economic reasons, whereas decentralized solutions might be favored when decentralized economic growth is at stake.

## Materials and methods

### Study area

**Fig 1:**
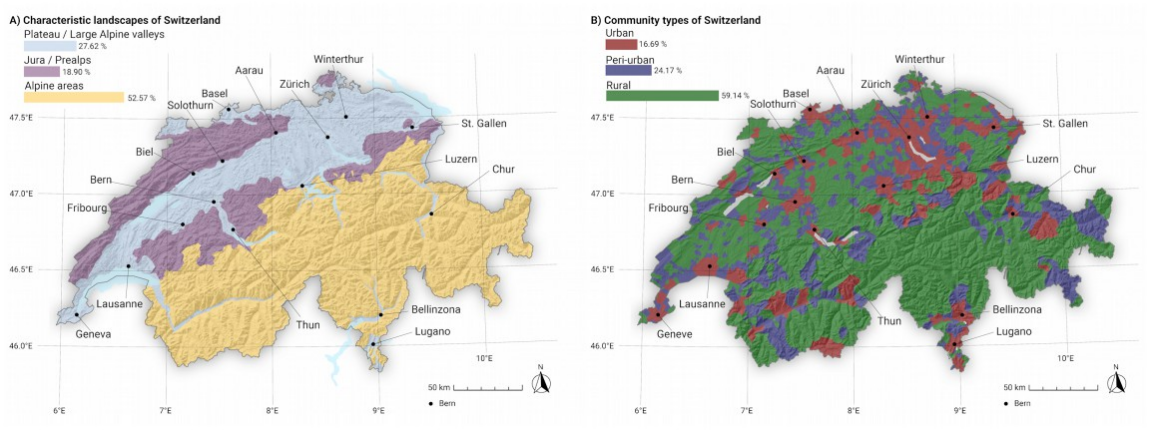
Main characteristic landscapes (A) and main municipality types of Switzerland (B). A: Landscapes on the plateau (blue), mid-elevation landscapes of the Jura and the Prealps (purple), and mountain landscapes of the Alps (orange). B: Municipality types of Switzerland are defined as urban (red), peri-urban (blue), and rural (green) and are derived from the official nine-category municipality typology based on socio-economic and spatial characteristics [22]. Swiss digital elevation model is provided by swisstopo DEM25/200m [23], while Swiss national borders are provided by swisstopo [24].

Switzerland is a Central European country that has a well-developed landscape policy and considers a broad variety of landscapes an important asset for health and place attachment of the population and as a basis for tourism. There are three main types of landscapes in Switzerland [20] (see Fig 1): The flat plateau category encompasses much of the north-eastern and south-western region of the country, as well as large valley bottoms in the Alpine region, and is characterized by settlements and agricultural production reaching up to 700 m a.s.l. The second category encompasses the mid-elevation mountainous areas of the Jura and Prealps regions, with moderate slopes, small valleys, and extensive forests that reach up to 1200 m a.s.l. The third category encompasses the Alpine region, which features steep slopes, high elevations ranging from 1200 to >3000 m a.s.l., stony terrain, and deglaciated zones. It also includes touristic villages situated in high inner-alpine valleys. About 70% of Switzerland is characterized as mountainous and about 30% as flat to hilly [21]. The Jura and Alps mountain ranges are separated by the plateau, which is home to a large part of the country’s eight million inhabitants.

According to main Swiss municipality types [22] urbanized municipalities of Switzerland account for about 17% of the country’s land area but are where 63% of the population reside and where 75% of the workforce are employed. In contrast, rural municipalities, which make up 59% of the area, are home to only 16% of the population and 10% of the workforce. Finally, peri-urban municipalities host 21% of the population and 15% of the workforce on 24% of the country’s land area.

### Modeling approach using Marxan

This study used a widely known as an open source optimization package (Marxan) frequently applied in conservation studies but also with a known record in related fields [11, 25–27]. Marxan is a popular solver that seeks to identify optimal solutions for conserving biodiversity while minimizing costs. Marxan itself is not a mathematical model in the traditional sense, it employs mathematical optimization techniques to solve the usually conservation-related planning problem. It uses a simulated annealing algorithm, a type of metaheuristic optimization algorithm, to find the best spatial configuration of areas that meets predefined targets and minimizes costs or other objectives [11, 28, 29]. The objective function to maximize energy output is to minimize ecological and societal costs following score [11]:

$$Maximize\left(\sum_{i=1}^{n}\;\left(E_{i}\;\times \;\chi_{i}\right)\right)≙\;minimizing\left(\sum_{i=1}^{n}\;\left(C_{i}\;\times \;\chi_{i}\right)+\sum_{i=1}^{n}\;\left(S_{i}\;\times \;\chi_{i}\right)\right)$$ (1)

$$\sum_{i=1}^{n}(E_{i}\times \;\chi_{i})=\text{Energy}\;\text{output}$$

$$\sum_{i=1}^{n}(C_{i}\times \;\chi_{i})=\;\text{Ecological}\;\text{costs}$$

$$\sum_{i=1}^{n}(S_{i}\times \;\chi_{i})=\;\text{Social}\;\text{costs}$$

*x =* a binary variable indicating whether planning unit *i* is selected for energy production (1) or not (0).

n = the total number of planning units considered

E_i_ = energy output potential of planning unit *i*

C_i_ = ecological costs associated with planning unit *i*

S_i_ = social costs associated with planning unit *i*

**Fig 2:**
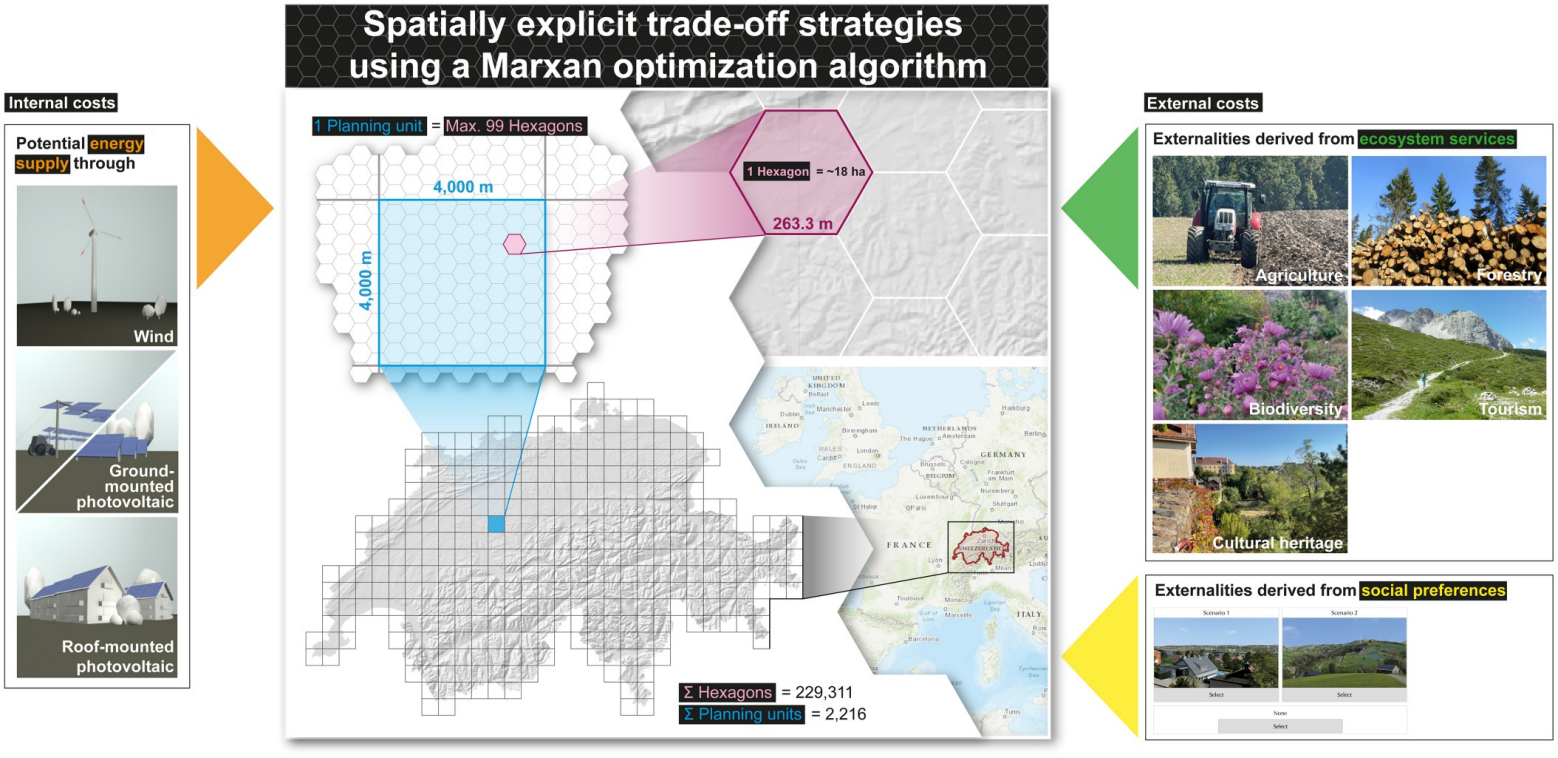
Overview of the modeling approach using Marxan optimization algorithm. Internal costs (energy supply) on the left side and external costs (externalities from ecosystem services and society) on the right side are shown as inputs for trade-off scenario development. The model is based on defined planning units (PUs) of 4 × 4 km squares (n = 2216), each consisting of up to 99 hexagons. European background map is provided by Leaflet (https://leafletjs.com/) | Tiles © Esri—Esri, DeLorme, NAVTEQ, TomTom, Intermap, iPC, USGS, FAO, NPS, NRCAN, GeoBase, Kadaster NL, Ordnance Survey, Esri Japan, METI, Esri China (Hong Kong), and the GIS User Community. Swiss digital elevation model is provided by swisstopo DEM25/200m [23], while Swiss national borders are provided by swisstopo [24].

Götz [30] describes the primary advantage of this algorithm as its ability to escape local minima. Thus, by permitting these stochastic unfavorable moves, it prevents becoming trapped in local positions. However, solvers other than Marxan, such as Gurobi, COIN-OR, Zonation, and IBM CPLEX, are also used for different types of optimization problems. They differ in their underlying algorithms, features, and performance. Marxan operates on so-called spatially explicit planning units (PUs) where a given output–in our case energy–is generated at minimal ecosystem service costs and social costs. Costs were subdivided into internal costs (energy data, see section 7.3) and external costs (socio-ecological conflicts of energy production, see section 7.4). All costs were aggregated to the level of PU, as shown in Fig 2, which provides a schematic overview of the model design. External social costs represent an evaluation of the perceived landscape quality within a certain dimension, and Jaeger et al. [31] stated an upper limit of perception distance to the surrounding landscapes. This dimension was set to the level of PU at 4 × 4 km (~2.48 × ~2.48 mi). Further, each PU consisted of up to 99 hexagons, each potentially hosting one wind turbine or numerous PV installations on roofs (building-mounted, rm-PV) and in open space (ground-mounted, gm-PV). Hexagons are commonly used in spatial statistics and modeling, due to their tessellating properties, uniform coverage and connectivity, and suitability for spatial analysis techniques [32, 33].

### Energy data (internal costs)

#### Wind resource potential

The wind energy potential was calculated top–down according to Angelis-Dimakis et al. [34]. Unsuitable areas were successively eliminated for reasons related to physical characteristics (e.g. topography, elevation), planning regulations (e.g. distance to settlements, protected nature conservation areas, landscape protection areas) or land use (e.g. agricultural areas). As the starting point for this analysis an existing *annual average wind speed* map interpolated from meteo station data and calculated for 150 m above ground [35] was used.

*Physically suitable areas for wind turbines* were determined based on the methodology used by Huber et al. [8], Hergert [36], and Kienast et al. [37]. All areas with a mean annual wind speed >4.0 m/s (150 m above ground), stable soil, and slope <20% were included. Lakes, military areas, and all strictly non-negotiable protected nature conservation and landscape protection areas, e.g. mire landscapes of national importance, core zones of the Swiss National Park, or UNESCO World Heritage were excluded. Further all settlements with a buffer of 300 m (federal regulation) were excluded, too. Accessibility was considered by including areas within a buffer of 150 m from any road wider than or equal to 3 m and from any railway track.

The *power output* was calculated for Vestas V-112 (3,075 MW) wind turbines (Vestas, Aarhus N, Denmark), which perform well in low wind conditions, such as those present throughout much of Switzerland. In the hilly Swiss terrain, these turbines are often placed at hub heights of around 90 m and have a rotor diameter of 112 m. Suisse Eole’s web-based tool (https://wind-data.ch/tools/powercalc.php) was used to calculate power outputs based on continuous *average* wind speeds between 4.0 and 8.0 m/s (the highest annual average in Switzerland) and capacity factors, full load hours, and operating hours typical for Switzerland.

#### Ground-mounted and roof-mounted PV infrastructure

*Roof-mounted PV infrastructure*. The maximum potential solar energy produced by PV panels mounted on roofs was determined using modeled data from sonnendach.ch [38]. For each roof (reference year 2015) larger than 10 m^2^, *roof geometry*, *shading*, and *maximum potential radiation* was analyzed, and an *annual average energy yield* was assigned. Based on this yield, each roof was assigned a quality class between 1 (low, <800 kWh/m^2^/a) and 5 (excellent, >1400 kWh/m^2^/a). Subsequent analyses focused on the three top classes (excellent = class 5; very good = class 4; good = class 3, 1000 kWh/m^2^/a).

*Open-space ground-mounted PV*. Using the Swiss land survey from 2004–2009 [39], all green and impervious areas (such as arable land, meadows, scrubby forest and bushes, motorway/railway greenery, and parking areas) that are not strictly protected as areas potentially eligible for open-space gm-PV were extracted. Only areas within a buffer of 150 m from roads ≥3 m wide and from any railway track, and areas outside strictly protected areas (see section 6.3.1) were included. 1 MWp per 1.4 ha and 900 sun hours per year was assumed, which translates to 640 MWh/a/ha on average. Given the numerous high-elevation sites in Switzerland, with shading and relatively steep slopes, this conservative value is deemed justified.

#### Generating the spatially explicit energy input for optimization

The Marxan optimization software needs georeferenced planning units. A grid with regularly spaced 4 × 4 km squares was defined as planning units (PUs), for which the potential energy from wind, rm-PV, and gm-PV was calculated. Each PU consisted of a maximum of 99 hexagons (edge length 263.3 m, diagonal length 526.5 m), each of which could potentially host 1 wind turbine. The latter assumption is based on recommendations from Lütkehus et al. [40] and Huber et al. [8] regarding the minimum distance between wind turbine sites. Turbines were only allowed when the hexagons were uninhabited by humans and the hexagon center was >300 m away from a settlement (federal regulation).

To determine the energy output per 4 × 4 km PU, first the most energy-rich hexagons for wind energy in the PU were selected and summed their potential energy outputs. A maximum of 10 (15 for the Jura region) turbine sites per PU was allowed. Concerning rm-PV, for each PU the energy output from roofs in all hexagons was summed. To calculate the energy output from gm-PV in a given PU, a maximum of four hexagons that were the most energy-rich in terms of gm-PV was selected and their energy output was summed. Finally, the potential energy outputs from wind, rm-PV, and gm-PV was summed to estimate the total potential energy production per PU.

### Socio-ecological conflicts of energy production (external costs)

Potential conflicts between energy production and the environment were considered in two ways:

- *Potential conflicts that cannot be negotiated*: All strictly protected areas and infrastructure (e.g. airport clearance areas) were eliminated as potential zones for energy production prior to optimization.
- *Negotiable potential conflicts*: All areas without strict regulations were included in the optimization and flagged with the corresponding external costs, e.g. biodiversity loss or population reactance.

Two types of external costs were considered: ecosystem services and social preferences.

#### Externalities derived from ecosystem service calculations

This study used the approach of ecosystem service calculation applied by Huber et al. [8], Kienast et al. [7], and Egli et al. [10], as well as Hastik [5] and Jongbloed et al. [41]. External costs were assessed for negotiable conflicts with provisioning, regulating and cultural services, subdivided into the categories agriculture, forestry, mixed land uses for food and timber production, biodiversity, tourism, and cultural heritage. *Ecosystem service classification* was based on the current Common International Classification of Ecosystem Services (CICES V4.3) terminology presented by the European Environment Agency [42] and the spatialized data used by Kienast et al. [7] and Egli [10]. Externalities were expressed in ‘external cost units’ and not in convertible currency (ECU_ess_ for external costs involving ecosystem services and ECU_soc_ for external costs involving social preference/reactance). These ECUs were generated with look-up tables and transformed into ordinal values between 0 (no externalities) to 4 (high externalities).

*Externalities involving agriculture*, *forestry*, *and miscellaneous land uses*. There is some competition between sustainable energy production and agricultural and forestry products. They are minor for wind energy but result from local physical impacts during wind turbine construction and operation (concrete foundation, dense network of access roads). Ground-mounted PV infrastructure on e.g. open fields generates considerable externalities, as there is a clear reduction in yield or even a complete loss for most crops, depending on the design of the PV construction (e.g. heights of the PV panels). However, as recent research shows, some crops (e.g. potatoes) actually show greater yields when heavily shaded [43, 44]. In these cases, our externality calculations would be too conservative. S1 Table contains information on the applied externalities.

*Externalities involving biodiversity*. The potential externalities of wind turbines (mainly bat and bird collisions) were calculated using spatial data from Huber et al. [8]. This dataset includes inventories, such as a map of occurences of breeding and visiting birds and of bird sanctuaries designated for migratory and waterbirds [45], a model-based bird sensitivity map [46], amphibian spawning areas of national importance, nationally important floodplains, dry grasslands of national importance, Emerald areas, federal game reserves, forest reserves, and Ramsar sites. Each of these indicators were weighted equally, and added and re-scaled to a value (ECU_ess_) between 0 and 4, sampled at a 1 ha resolution. The potential externalities of gm-PV related to biodiversity, mainly biodiversity loss in species-rich habitats, are listed in S1 Table.

*Externalities involving tourism*. Switzerland is dependent on foreign and domestic tourism and outdoor activities rank high in the population preferences. Hence, our trade-off tool assumes that decision-makers in the tourism sector try hard to avoid visual impacts in the landscape caused by windfarms and gm-PV. This increases the externalities for energy infrastructure in outdoor tourism areas where an unobstructed visual landscape is prioritized. To map these areas, the maps of national park and nature parks were used, along with the Federal Inventory of Sites of special landscape beauty [47]. Further, a Swiss-wide map of the potential for nearby recreation [46] was used. As indicators of tourism activity, maps showing mountain bike trails and ski trails (Switzerland Mobility; [48] and overnight stays in hotels [49]) were additionally used. Values from these various input data were weighted equally, summed, re-scaled to 0–4 ECU_ess_, and gridded to 1 ha.

*Externalities involving cultural heritage*. Externalities of PV *or* wind energy infrastructure related to cultural heritage arise mainly for objects that are protected under national or regional heritage acts. A cost map was created by summing the following data layers: (1) areas of natural and cultural importance identified by UNESCO [50]; (2) buildings or structures that define culturally important zones, such as castles, churches, and historical landmarks [51]; and (3) the Federal Inventory of Swiss Cultural Monuments [52]. Point data were buffered with 300 m. Depending on the importance of the building or inventory, additive “cost values” were assigned that were scaled to 0–4 ECU_ess_.

#### Social externalities derived from social preference values

The second type of externality was used to capture people’s preferences related to landscape developments triggered by REI. It stems from a Swiss-wide representative online panel survey where respondents (n = 1062) performed a visual choice experiment resulting in “choices” and reflecting stated preferences [53]. The inverse of their preference values was used to express reactance to renewable energies in specific landscape contexts. High reactance towards a project translates easily to high project costs, due to delayed permits and authorization of the project.

Preferences of the Swiss population towards renewable energy installations were determined by means of a representative online panel survey using a standardized survey instrument. The study received ethical approval from the Ethics Committee at ETH Zurich on March 7th, 2018. The committee members involved in the approval process were Lutz Wingert, Christoph Höscher, Matthias Mahlmann, Marino Menozotti, Kai-Uwe Schmitt, Michael Siegrist, William R. Taylor, and Peter Wolf. The study was assigned the approval number EK 2017-N-69. The market research institute and online panel provider BILENDI GmbH was commissioned to perform the survey that the authors created. The survey was conducted between November 2018 and March 2019 following a pre-test. A total of 1062 people took part in the online panel survey. At no point were the identities of the participants known to the research team as this information had already been anonymized by the panel provider before being provided.

The sample is representative for Switzerland in terms of language, age, gender, education and designated landscape type. Details about the study sample, the sampling procedure, the questionnaire, and the choice experiment component are described in Salak et al. [53, 54], and data are publicly available at an online repository [55]. Therefore, the integration of the stated preference data into the optimization is detailed primarily. The core element of the survey instrument is a so-called visual discrete choice experiment. In this experiment a selection of different scenarios is presented to the respondents for decision. Each scenario consists of a combination of attributes and their characteristic levels. The following attributes were considered: “landscape” (LS), “wind infrastructure” (W) and “photovoltaic infrastructure” (PV).

The attribute “landscape” consists of eight landscapes that are characteristic of Switzerland (Table 1). Together with a group of experts, the specific landscapes were selected based on the main biogeographic regions of Switzerland, in a phase preceding the survey [56]. These regions are visually distinguished by the extent of urbanization and the roughness of their topography.

**Table 1:** Description of typical Swiss landscapes used in this study. The “Characteristic landscapes” column refers to characteristic landscapes used as attributes in the discrete choice experiment of the national representative online survey [53, 54, 56]. These characteristic landscapes are upscaled to larger landscape units displayed in the “Name” column (see also Fig 1).

No.	Name	Characteristic landscape	Description	Coverage (km^2^)	Coverage (%)
**1**	**Plateau**	**PLAT_URB**	Settlement-dominated plateau	3848.21	9.32
	**Plateau**	**PLAT_AGRI**	Agriculture-dominated plateau	4957.71	12.01
	**Plateau**	**ALP_URB**	Mountain valleys characterized by dense settlements and infrastructure	691.15	1.67
**2**	**Mid-elevation mountainous areas**	**PRE_ALPS**	Northern Alpine mountainous areas at medium elevation with dominant cattle grazing and farm infrastructure	3912.39	9.48
	**Mid-elevation mountainous areas**	**JURA**	Gently rolling Jura hills	3890.53	9.42
**3**	**High-elevation mountainous areas**	**ALP_TOUR**	Touristic areas in the mountains with clear tourism infrastructure	4435.21	10.74
	**High-elevation mountainous areas**	**ALP_INF** ^a^	Central Alpine mountainous areas at medium/high elevation with dominant cattle grazing and farm infrastructure	7651.99	18.53
	**High-elevation mountainous areas**	**ALP**	Rather pristine mountainous areas at high elevation with very little infrastructure	9620.33	23.30
**-**	**-**	**INNER CITIES**	excluded	1909.51	4.62
**-**	**-**	**LAKES**	excluded	370.71	0.90

^a^Landscape ALP_INF was interpolated, as it was not designed as an attribute in the discrete choice experiment of the survey.

Alpine landscapes with infrastructures (ALP_INF) derived from the evaluation of pre-alpine landscapes (PRE_ALPS), as there are many similarities between these landscape types that warrant further consideration. Further, evaluations related to power lines, which were an attribute in the choice experiment but were not relevant for choice simulations in this study, were excluded.

The attribute “PV” includes not only rm-PV, but also gm-PV systems, as these could become increasingly important in the future. In total, the combination of the attribute levels results in 128 possible scenarios (8 landscape levels * 4 wind attribute levels * 4 PV attribute levels = 128). A so-called D-optimal (efficient) fractional factorial minimal overlap design was used, in which each respondent was confronted with 15 different decision situations (choice tasks). The presented scenarios were “unlabeled” in that the attribute levels were presented purely visually without further textual explanation, although explanations were provided in the introduction section prior to the discrete choice experiment. Each choice task presented to the respondent included two alternatives (= two different scenarios) per decision and one “neither” option (opt-out). Cleaning procedures [54] resulted in a total of 844 respondents providing 12,660 choice observations (15 choice tasks * 844 respondents = 12,660 choices).

**Fig 3:**
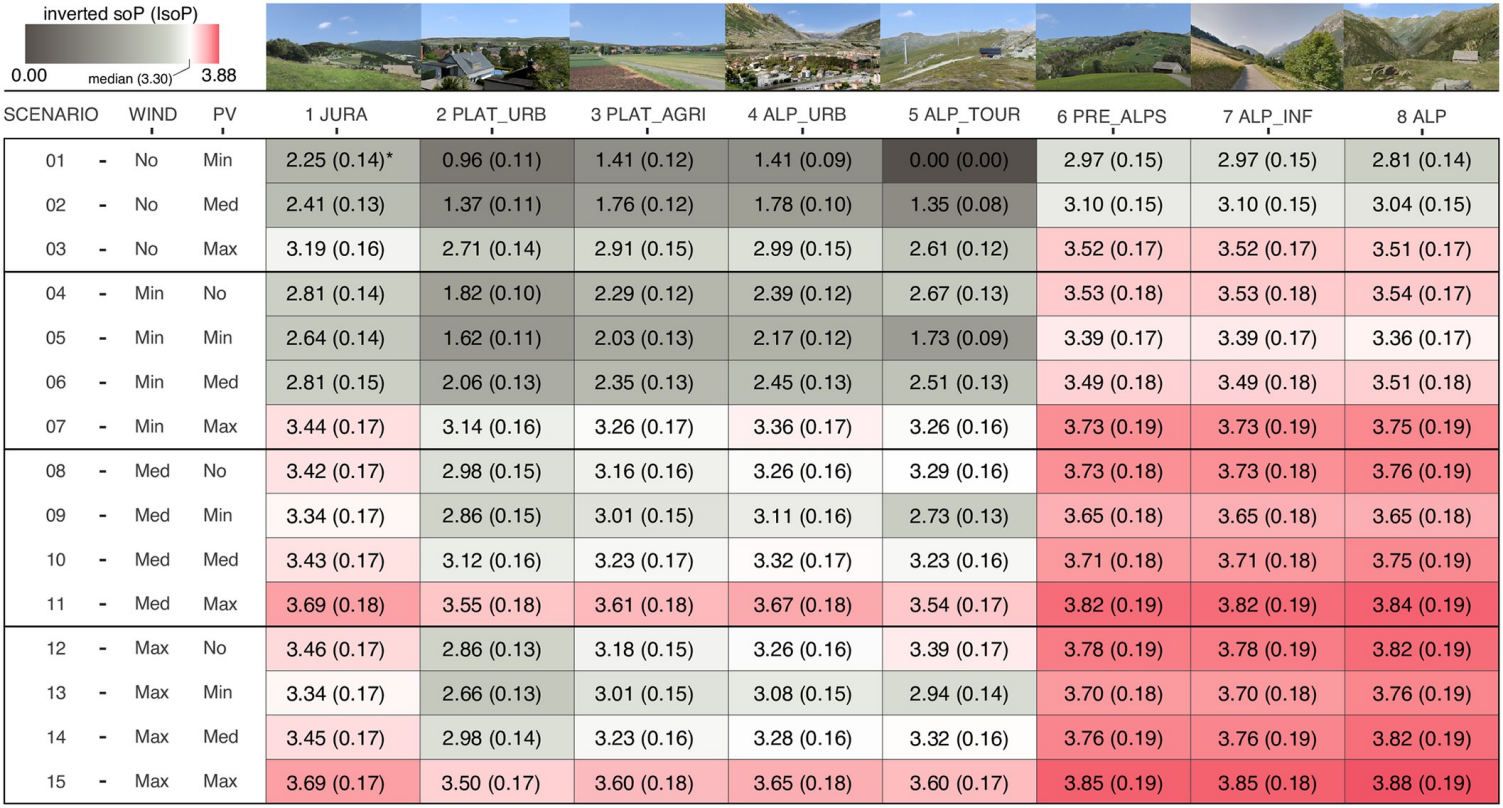
Social preferences of renewable energy scenarios in various landscapes. It shows the Inverted share of preference (IsoP), with standard error (SE), per characteristic landscape type (n = 1, 8) and scenario (S = 1, 15) of the choice simulation expressing external costs related to social preferences of renewable energy infrastructure (REI) [53]. A higher IsoP value indicates higher costs. IsoP can also be interpreted as the cost of reactance. This study applied the formula IsoP(n,S) = SoP_max_−SoP(n,S), where IsoP(n,S) = mean inverted share of preference of landscape type n in scenario S; SoP_max_ = maximum absolute share of preference; SoP_(n,S)_ = share of preference of landscape type n in scenario S (1 ECU_soc_ = 1 IsoP).

Following Allenby et al. [57] and Lenk et al. [58], first a multinominal logit hierarchical Bayes analysis (MNL-HB) was computed based upon choice observations, resulting in individual preference profiles for each respondent. Those profiles were used as input for a randomized first choice (RFC) simulation (5925 iterations per respondent, including “none” option), resulting in “shares of preferences” (SoP) for each scenario. Adherent to Orme [59], SoP represented the average interest of the respondents to the various scenarios. The inverted SoPs (IsoPs) were used to express the social externalities associated with a given scenario (Fig 3).

#### Generating the spatially explicit externalities (costs) for optimization

To calculate the external costs of the potential energy mix per 4 × 4 km square PU, all corresponding external costs were summed at the spatial resolution of the PU.

For external costs involving ecosystem service calculations, the costs in ECU_ess_ were summed for all hectares with REI, aggregated them to the hexagon, and finally to the PU. For spatially explicit social externalities, the potential energy mix (wind, PV) for each PU was calculated and matched with the responses from the choice experiment. This matching (S1 and S2 Tables) was conducted by translating each REI mix of the choice experiment into an REI attribute in the PU. Once a match was found, the social costs in ECU_soc_ were assigned using the inverted share of preference (IsoP) from Fig 3.

### Marxan parameterization

#### Model input

Energy data and externalities were used to generate the input data for Marxan. Marxan is usually used as a conservation planning tool, but the idea of the program remains the same for this application: select the best features at the lowest costs to meet a certain target. Marxan requires three sets of input variables [28]:

1. “features” representing the benefit (here energy) offered by the PU. In our case the PU is the 4 × 4 km square; a total of 2216 PUs were eligible to be used in our optimization, with a total potential energy supply of 170 TWh/a when including gm-PV and 114 TWh/a when excluding gm-PV.
2. “feature targets,” the envisaged total benefit (here energy), over all PUs in the optimization. In our case this value is 25TWh/a in all strategies.
3. the “cost” associated with including a PU in the solution. The sums of environmental costs ECU_ess_ and ECU_soc_ were normalized [0;1] per 4 × 4 km PU. Therefore, ecosystem services (ESS) and social preferences (SOC) had the same weight and contributed equally to the optimization procedures.

With all the inputs in place, a typical Marxan run consisted of 100 repetitions/run with 1,000,000 iterations, where PUs were randomly added and removed to maximize benefits (reaching the target as closely as possible) while minimizing costs over the entire system. The following three planning strategies were run:

1. [NRG]: seeking pure energy effectivity by selecting sites with the highest energy yields (high energy effectivity) without considering external costs.
2. [ESS]: selecting the sites with the highest energy yields while maintaining the lowest possible ecosystem service costs.
3. [SOC]: selecting the sites with the highest energy yields while maintaining the lowest possible social costs.

Because the impact on the visual quality of landscapes is distinctly different when gm-PV is used compared with when rm-PV is used, each strategy was flagged with the label “-OS” (for open space) if gm-PV contributed to the energy production.

#### Model outputs

Marxan generated various outputs for a series of runs (here 100):

- The solution for each run.
- The “best” solution, i.e. the one with the absolute lowest costs out of all runs.
- A list of how frequently individual PUs are chosen.

Whilst the first and second outputs are “crisp solutions” with an exact number of sites, the third output indicates their irreplaceability over repeated runs.

## Results

Assessing the efficiency of strategies can be done by considering three key dimensions represented by the following efficiency triangle:

- *Spatial efficiency*, expressing how much land is overbuilt (expressed in planning units) and whether the location of the selected planning units is dispersed or clumped.
- *Environmental efficiency*, expressing how high the environmental costs are per unit of produced energy.
- *Social efficiency*, expressing how high the social costs are per unit of produced energy.

### Strategy snapshot

**Fig 4:**
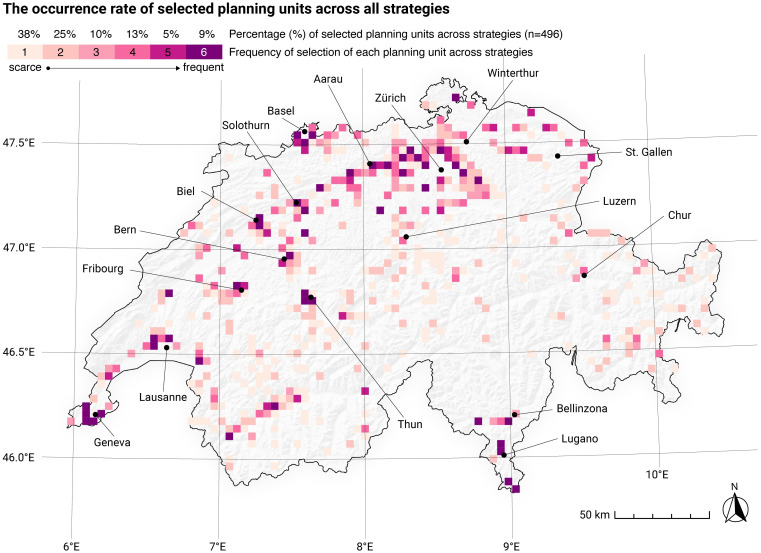
Overview of the frequencies of selected planning units (Pus) across the applied scenarios. 9% of all selected PUs are selected by every applied trade-off strategy (stable PU), while 38% of the selected PUs are selected for only one specific strategy (unstable PU). Swiss digital elevation model is provided by swisstopo DEM25/200m [23], while Swiss national borders are provided by swisstopo [24].

The geographical distribution and frequency of selected planning units (Fig 4) provide insights into centralization and decentralization tendencies of the potential future energy system. It shows indispensable sites (9%),, as well as many sites that are negotiable depending on the strategy. Planning units on the plateau and in the larger inner-alpine valleys of the Alps are selected more frequently and thus can be considered more indispensable for energy transformation than ones on the Jura ridges, in the Prealps, and in Alpine landscapes. Planning units located in the vicinity of urban areas are particularly important for energy transformation, as they are commonly selected across all strategies. Conversely, units located in mid- and high-elevation mountainous regions may only be chosen if no other options are available.

**Fig 5:**
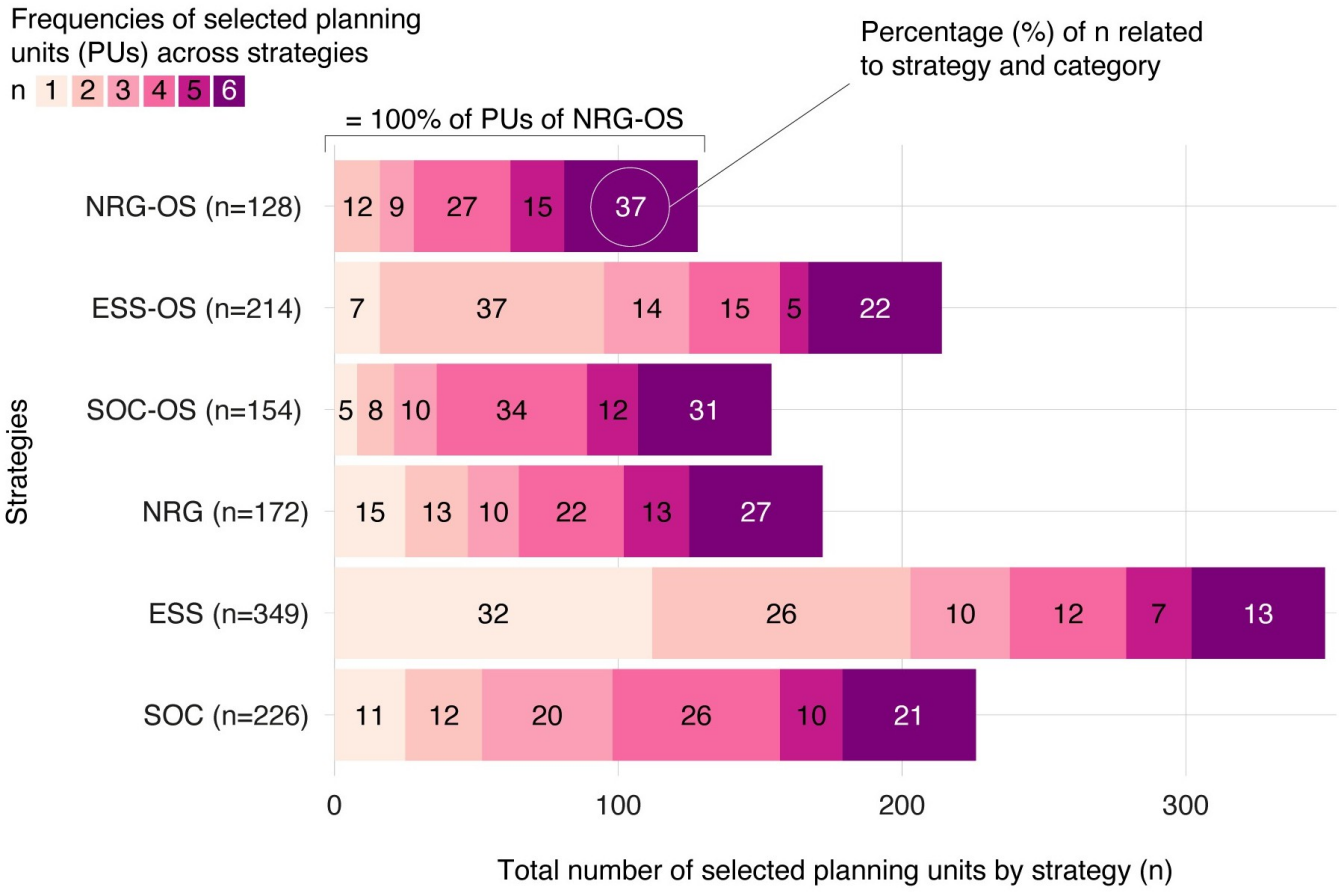
Overview of the frequencies of selected planning units (PUs), by strategy, in percentage. 37% of the selected planning units of strategy NRG-OS consist of PUs that are also selected by all other trade-off strategies.

To better understand the implications of the strategies on PU selection, reference is made to Fig 5. It is evident that strategies that focus on energy yield optimization per PU (NRG-OS, NRG) have more indispensable sites, i.e., sites selected with higher frequency across the strategies. Strategies that focus on ecosystem service cost optimization (ESS-OS, ESS), on the other hand, rely on PUs with low ecological impacts but at the same time relatively low energy outputs, which significantly increases the number of PUs. Many of the selected PUs are chosen for this strategy only. In terms of PU selection, strategies that focus on social cost optimization (SOC-OS, SOC) lean more toward energy efficiency strategies than environmental cost optimization strategies, by using many cross-strategy PUs to reach the required amount of energy.

**Fig 6:**
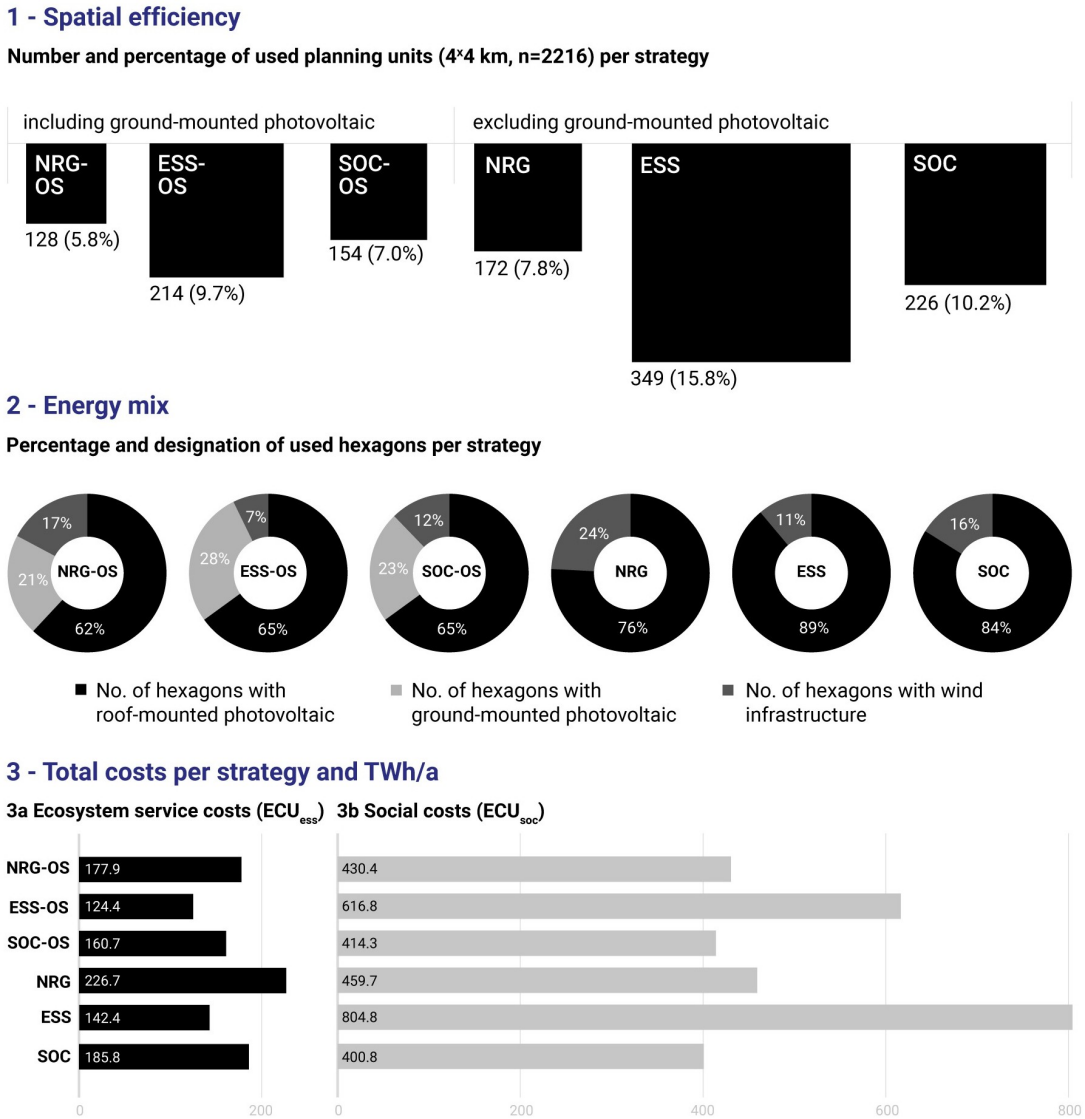
Details of optimization results per strategy with and without ground-mounted photovoltaics (gm-PV). Spatial efficiency (1) indicates the usage of PUs per strategy in comparison to all available PUs (%). It is assumed that fewer PUs selected per strategy to fulfill the desired energy output (25 TWh/a) corresponds to higher spatial efficiency. Energy mix (2) indicates the designation of hexagons per strategy and provides an overview of the potential energy mix if the strategy is implemented. Ecosystem service costs (3a): the fewer ecosystem services that are lost, the better the ecosystem service efficiency (the lower the costs). Social costs (3b): the fewer social costs related to people’s preferences in energy landscape development, the better the social efficiency (the lower the costs).

A direct comparison of strategies using our proposed efficiency triangle shows that using open-space ground-mounted PV is–for all strategies–spatially, ecologically, and socially more efficient than omitting gm-PV (Fig 6). Strategies that select sites without considering ecosystem service costs and social costs (NRG-OS, NRG) select PUs that have high ecosystem service costs and surprisingly low social costs. Strategies with a focus on low ecosystem service costs (ESS-OS, ESS) optimize costs by reducing wind energy considerably. This avoidance leads to very large numbers of PUs in landscapes that are not considered acceptable by the population, which results in low social efficiency (high social costs). By contrast, strategies optimized for the lowest possible social costs (SOC-OS, SOC) perform well across all categories and outperform other optimization strategies in social aspects. Further, it highlights the overall good performance of strategy SOC-OS and shows that if social costs are minimized, costs for ecosystem services are also considerably lowered, in particular compared with state-of-the art technocratic-economic optimization models represented by strategies NRG-OS and NRG.

Strategy NRG-OS is spatially most efficient, yielding the smallest possible number of PUs to generate the desired energy amounts. High-energy output cells with PV in peri-urban areas are prioritized and supplemented with wind and gm-PV sites. This optimization results in the best energy efficiency per PU and a mix with the lowest percentage of rm-PV or gm-PV infrastructure, but the highest percentage of wind energy infrastructure. While it has low social preference costs, the overall ecosystem service costs are very high.

When aiming for the lowest ecosystem service costs (ESS-OS), the algorithm excludes many energy-rich sites with high costs, particularly windfarms. Instead, potentially less environmentally harmful infrastructure, like rm-PV and gm-PV, is selected. ESS-OS is effective for reducing ecosystem service costs, but it incurs high social costs and is spatially inefficient, due to the heavy use of gm-PV and reduced use of wind turbines.

While strategy SOC boasts the lowest overall social costs, strategy SOC-OS is significantly superior in terms of spatial costs and ecosystem service costs while remaining similarly efficient in terms of social costs. It achieves remarkable spatial and social efficiency overall, with only moderate impacts on ecosystem service costs. Hence, strategy SOC-OS shows promising solutions in all analyzed categories, representing a surprisingly good strategy environmentally.

The costs differ significantly between the strategies in 26 out of 30 pairwise comparisons (Table 2). Strategy NRG-OS incurs the highest ecosystem service costs and social costs per PU but requires the smallest number of PUs to attain the desired energy output. On the other hand, ESS performs well in both cost categories but requires the largest number of PUs. Meanwhile, SOC-OS ranks in the middle in both ecosystem service costs (lower than NRG-OS and NRG, but higher than SOC, ESS-OS, and ESS) and social costs (lower than NRG-OS and ESS-OS, equal to NRG, but higher than ESS and SOC), while requiring the second smallest number of PUs to generate the desired energy amount.

**Table 2:** Group differences of strategies per planning unit (PU) and by external costs. A TukeyHSD (honestly significance difference) single-step multiple comparison procedure was applied to find means that are significantly different from each other with respect to differences in group sizes.

Group comparison	Group differences related to…							
	…ecosystem service costs				…social costs			
	Est.	CI_low_	CI_high_	adj. p-value	Est.	CI_low_	CI_high_	adj. p-value
ESS-SOC	-0.414	-0.545	-0.283	≥ 0.001***	0.532	0.322	0.743	≥ 0.001***
NRG-SOC	0.496	0.340	0.651	≥ 0.001***	0.899	0.649	1.149	≥ 0.001***
SOC-OS-SOC	0.221	0.061	0.382	≥ 0.001***	0.916	0.659	1.174	≥ 0.001***
ESS-OS-SOC	-0.241	-0.387	-0.094	≥ 0.001***	1.108	0.873	1.344	≥ 0.001***
NRG-OS-SOC	0.568	0.398	0.738	≥ 0.001***	1.589	1.316	1.862	≥ 0.001***
NRG-ESS	0.910	0.767	1.053	≥ 0.001***	0.367	0.137	0.597	≥ 0.001***
SOC-OS-ESS	0.635	0.487	0.784	≥ 0.001***	0.384	0.145	0.623	≥ 0.001***
ESS-OS-ESS	0.173	0.040	0.307	> 0.003**	0.576	0.362	0.790	≥ 0.001***
NRG-OS-ESS	0.982	0.823	1.141	≥ 0.001***	1.057	0.802	1.312	≥ 0.001***
SOC-OS-NRG	-0.274	-0.445	-0.104	≥ 0.001***	0.017	-0.256	0.291	0.999
ESS-OS-NRG	-0.736	-0.894	-0.579	≥ 0.001***	0.209	-0.043	0.462	0.170
NRG-OS-NRG	0.072	-0.107	0.252	0.862	0.690	0.402	0.978	≥ 0.001***
ESS-OS-SOC-OS	-0.462	-0.624	-0.299	≥ 0.001***	0.192	-0.069	0.453	0.287
NRG-OS-SOC-OS	0.347	0.163	0.530	≥ 0.001***	0.673	0.378	0.968	≥ 0.001***
NRG-OS-ESS-OS	0.808	0.637	0.980	≥ 0.001***	0.481	0.205	0.757	≥ 0.001***
Ranking by external costs/PU	ECU_ess_: (high) NRG-OS > NRG > SOC-OS > SOC > ESS-OS > ESS (low)							
	ECU_soc_: (high) NRG-OS > ESS-OS > SOC-OS > NRG > ESS > SOC (low)							

Est. = estimate, CI_low_ = lower confidence interval limit, CI_high_ = upper confidence interval limit, Adj. p-value = adjusted p-value. Levels of significance:

*** p ≤ 0.001,

** p ≤ 0.01,

* p ≤ 0.05.

Gray shading indicates non-significant differences. At the bottom of the table a ranking of strategies from high to low costs by PU is shown. ECU_ess_ = energy cost unit related to ecosystem services, ECU_soc_ = energy cost unit related to social preference.

Since strategies including open-space gm-PV generally perform better than those without, our further analysis concentrates on strategies including this infrastructure.

### Potential effects on both ecosystem service costs and social costs

**Fig 7:**
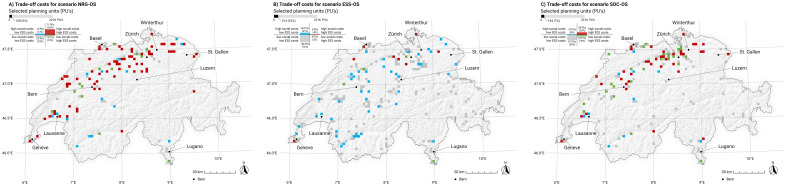
Spatial representation of the combined ecosystem service costs and social costs for the selected planning units (PUs) of all strategies using ground-mounted photovoltaic infrastructure (gm-PV). Each top bar represents the number of PUs required per strategy to fulfill the desired energy goal of 25 TWh/a. The PUs are classified by their quantile into four classes representing (1) low ecosystem service (ESS) costs and low social costs (grey), (2) low ecosystem service costs and high social costs (green), (3) high ecosystem service costs and low social costs (blue) and (4) high ecosystem service costs and high social costs (red). Swiss digital elevation model is provided by swisstopo DEM25/200m [23], while Swiss national borders are provided by swisstopo [24].

Each strategy is characterized by a ratio of ecological and social costs. Fig 7 illustrates the spatial cost pattern of each strategy by distinguishing the number of “expensive” (high costs) and “inexpensive” (low costs) PUs.

Strategy NRG-OS boasts the smallest number of PUs, but this comes at a high overall external cost. More than three-quarters of the selected PUs are associated with high social costs and/or ecosystem service costs, leaving only a small percentage of PUs with low external costs. More than half of the PUs are associated with both high social costs and high ecosystem service costs.

Strategy ESS-OS results in a significant increase in the total number of selected PUs compared with NRG-OS. However, it also has a smaller number of PUs affected by high external costs, with about three-quarters of the selected PUs having low ecosystem service costs and low social costs. While about one-quarter of the PUs have high ecosystem service costs and/or high social costs, only 4% have both high ecosystem service costs and high social costs.

Strategy SOC-OS has a comparable number of selected PUs to strategy NRG-OS, but significantly fewer PUs with high ecosystem service costs and high social costs (25% instead of 55%). Most of the selected PUs have low ecosystem service costs and low social costs. Overall costs indicate that these PUs are preferable according to social preference ratings, making them less costly from a social perspective than both ESS-OS and NRG-OS. Additionally, this strategy requires fewer PUs overall to achieve the desired energy production compared to ESS-OS, indicating higher spatial efficiency.

### Potential effects on characteristic landscapes

**Fig 8:**
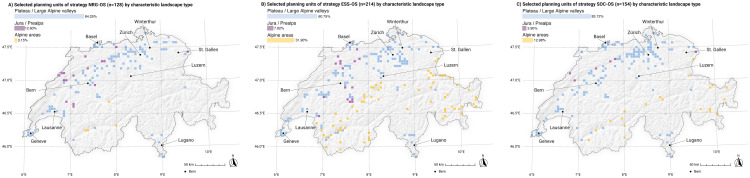
Selected PUs of strategies including ground-mounted photovoltaic infrastructure (gm-PV), separated by characteristic landscape type. NRG-OS = strategy focusing purely on energy effectivity by selecting sites with the highest energy yields (high energy production per year) without considering environmental and social external costs, ESS-OS = strategy selecting the best sites in terms of energy output at the lowest environmental costs, SOC-OS = strategy selecting the best sites in terms of energy at the lowest social costs. Swiss digital elevation model is provided by swisstopo DEM25/200m [23], while Swiss national borders are provided by swisstopo [24].

Landscapes on the plateau and larger Alpine valleys are the preferred landscapes for renewable energy infrastructure (REI) development in all strategies (Fig 8). High-elevation Alpine areas make up almost one-third of the PUs in ESS-OS, which is 10 times more than in NRG-OS and more than twice as much as in SOC-OS, meaning that they are almost completely spared in strategy NRG-OS. Mid-elevation mountainous regions, such as the Jura and Prealps, seem to generally play a minor role compared with other landscapes. However, strategy NRG-OS allocates one-eighth of its PUs to these landscapes, which is about double that in strategy ESS-OS and four times more than in strategy SOC-OS. In summary, in terms of characteristic landscapes, SOC-OS is comparable to NRG-OS but puts less pressure on the Jura and Prealps and also affects Alpine landscapes (less than ESS-OS).

### Potential effects on municipality types

**Fig 9:**
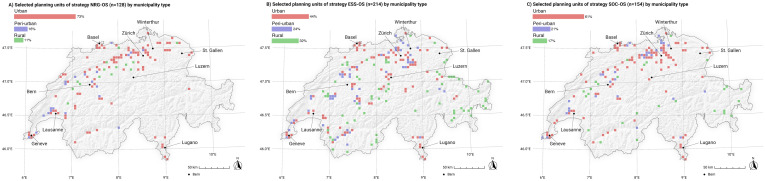
Selected PUs of strategies including ground-mounted photovoltaic infrastructure (gm-PV), separated by community type. NRG-OS = strategy focusing purely on energy effectivity by selecting sites with the highest energy yields (high energy production per year) without considering environmental and social external costs, ESS-OS = strategy selecting the best sites in terms of energy output at the lowest environmental costs, SOC-OS = strategy selecting the best sites in terms of energy at the lowest social costs. Swiss digital elevation model is provided by swisstopo DEM25/200m [23], while Swiss national borders are provided by swisstopo [24].

The strategies also differ in terms of the types of municipalities where the energy is sourced (Fig 9). While strategy NRG-OS is strongly bound to urban PUs (highest overall percentage), ESS-OS is more dispersed across all municipality types and is characterized as the least urban strategy (among the strategies including open-space gm-PV). Scenario SOC-OS fits in between these two strategies, as it relies on fewer urban PUs than NRG-OS but more than ESS-OS, and it uses more peri-urban and rural PUs than NRG-OS but fewer than ESS-OS. Differences between strategies including open-space gm-PV are especially evident in rural and urban areas, while peri-urban areas roughly level out in terms of their percentage per strategy (despite their differences in total number of PUs per strategy).

### Potential effects on the development of wind energy infrastructure

**Fig 10:**
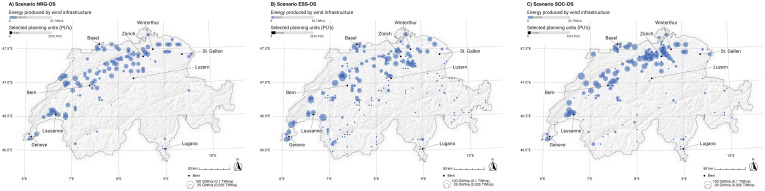
Spatial representation of wind-energy infrastructure in strategies including ground-mounted photovoltaic infrastructure (gm-PV). NRG-OS = strategy focusing purely on energy effectivity by selecting sites with the highest energy yields (high energy production per year) without considering environmental and social external costs, ESS-OS = strategy selecting the best sites in terms of energy output at the lowest environmental costs, SOC-OS = strategy selecting the best sites in terms of energy at the lowest social costs. Swiss digital elevation model is provided by swisstopo DEM25/200m [23], while Swiss national borders are provided by swisstopo [24].

Fig 10 provides a spatial overview of selected wind sites per strategy. When optimizing for the best sites independent of external costs (NRG-OS), powerful wind energy sites on the plateau and in the mid-elevation Jura mountains are selected. Many wind energy sites with a high electricity yield but considerable ecological costs are withdrawn from selection and replaced by a larger share of rm-PV when optimizing for a minimization of ecosystem service costs (ESS-OS). Further, many small, decentralized windfarms with a low ecological impact are observed in the high-elevation mountainous regions (small dots in the mountains).

Optimizing for the lowest social costs (SOC-OS) leads to almost no wind turbines in areas where people do not favor wind infrastructure, like in the Prealps mid-elevation mountainous regions and in the hilly landscape of the Jura. Some small wind farms are placed in the sensitive landscapes of the high-elevation Alps, as a result of the high energetic quality of the PUs. As seen before, the focus of this optimization strategy is clearly on landscapes on the plateau and inner-alpine valleys, which leads to a very concentrated development of REI on the foothills of the Alps.

### Potential effects on the development of -PV infrastructure

**Fig 11:**
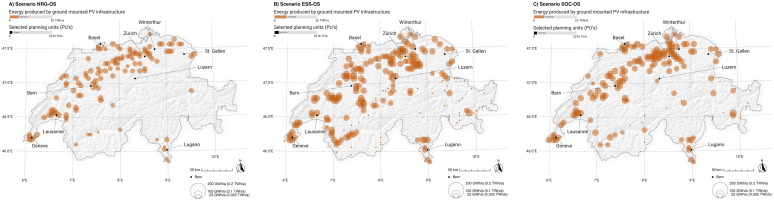
Spatial representation of ground-mounted photovoltaic infrastructure (gm-PV), separated by strategy. NRG-OS = strategy focusing purely on energy effectivity by selecting sites with the highest energy yields (high energy production per year) without considering environmental and social external costs, ESS-OS = strategy selecting the best sites in terms of energy output at the lowest environmental costs, SOC-OS = strategy selecting the best sites in terms of energy at the lowest social costs. Swiss digital elevation model is provided by swisstopo DEM25/200m [23], while Swiss national borders are provided by swisstopo [24].

Fig 11 completes the picture of potential landscape-driven developments through REI. As all strategies including open-space gm-PV rely on intense energy production based on this infrastructure (from 5.431 TWh/a to 7.017 TWh/a), it is unsurprising that in all strategies many large gm-PV installations are spread across the agriculture- and settlement-dominated plateau. This is rooted in both lower ecosystem service costs and social costs in these landscapes compared with in the Jura, Prealps and Alpine landscapes. Only ecosystem service optimization (ESS-OS) leads to the development of some low-energy (but still sufficient to be considered) gm-PV installations in Alpine regions. However, spatial concentration seems to differ across the strategies slightly. Strategy NRG-OS shows major developments in landscapes on the plateau and across the transition areas to the mid-elevation mountainous landscapes of the Jura, but only a little development in the inner-alpine valleys. Strategy ESS-OS leads to the development of multiple PV plants in the inner-alpine valleys and, as in the other strategies, around major cities. Strategy SOC-OS is primarily focused on landscapes on the plateau and on areas around major cities.

## Discussion

This study presents various strategies for generating 25 TWh/a of renewable energy, with the aim of optimizing REI development while minimizing negative impacts on ecosystem services and social aspects. Each scenario includes multiple types of renewable energy infrastructure, namely wind and PV. In the following discussion, first results are examined methodologically and second regarding the consequences of the strategies in an energy planning context.

### Methodological discussion

This work represents another promising implementation of the Marxan optimization approach outside its traditional field of application, building on work by Egli et al. [10] and Göke et al. [11]. This approach enabled the derivation of trade-offs between energy output, spatial efficiency, and ecological costs. Moreover, for the first time, it considered costs derived from representative social preferences to identify the most cost-effective solutions for any combination of the associated costs. This is a clear departure from Pareto-optimal approaches [60, 61] which only make sense for land-use trade-offs if the policy instruments are also evaluated [62], which explicitly was not included in this work in order to avoid limiting the scenarios. The Marxan method is open-source, flexible and scalable. It is widely applied and tested, but also shows some limitations in comparison to other solver algorithms. For example, it effectively conveys the idea that Marxan cannot optimize both ecosystem service costs and social costs simultaneously [60], as it is only possible to set one “cost layer” at a time, while other algorithms do include such features.

In the current study, the input layers have been considered equal and unweighted. Future research could involve assigning weights to these baseline data through a collaborative, interdisciplinary co-creative process, aiming to further enhance the accuracy of optimization results.

### Contextual discussion

The energy mixes presented in the results require contextual interpretation. They may change depending on technical aspects and spatial planning/policy constraints, or if data of different (more accurate) resolution are used. Further, they are sensitive to changes in the meaning of energy infrastructure through time which will be discussed in the following:

#### Technical and political aspects of wind energy production

Spielhofer et at. [63] have demonstrated that the choice of wind turbine type can significantly impact optimization results, particularly in high-elevation mountainous regions. Specifically, the use of adapted wind turbines with smaller rotor diameters and a lower mast base height can yield more favorable outcomes compared with using the same type of wind turbine across all regions. Further, Spielhofer et al. [63] have shown that minor changes in spatial planning policy, such as a relaxation of regulations regarding suitable sites (as in the case of forests), can have a significant impact on the spatial distribution and number of wind turbines, as this would lead to a centralization of wind energy development on the agriculture- and urban-dominated plateau. Consequently, the authors claim that a more restrictive planning policy leads to more wind turbines in the Alps. There are undeniable visual similarities in the spatial distribution of wind energy infrastructure in the present study and that of Spielhofer et al. [63], particularly with respect to strategy NRG-OS. However, this study observed a wider use of Alpine landscapes for wind energy infrastructure development only when optimizing for ecosystem services (ESS-OS), which is shown by Kati et al. [64] as a preferable scenario for a biodiversity-aware wind energy infrastructure development.

#### Electricity production in high-elevation mountainous regions

Kahl et al. [19] did show benefits of gm-PV infrastructure in high-elevation mountainous regions, especially regarding the winter season. In winter, high-elevation mountainous regions are characterized by fewer clouds and therefore clearer weather compared with the plateau, which favors electricity production through gm-PV. Liu and Stevens [65] support the siting of wind energy infrastructure in mountainous regions and preferably above hilltops due to performance reasons. Several studies show that the development of renewable energy facilities in high-elevation mountainous regions often lacks of social acceptance [53, 54, 66]. This present study confirms prior findings. It generally does not support the development of such infrastructure in high-elevation mountainous regions, as trade-offs clearly show high social costs and a lack of spatial efficiency. Consequently, this study supports developing REI in the surroundings of larger urban areas on the plateau and avoiding mid- and high-elevation mountainous regions whenever possible, even areas with existing touristic infrastructure.

#### Data resolution

While many sources of spatial information on ecosystem services were included in this study, future research could (a) further improve their spatial resolution and (b) work with a more refined and disaggregated classification of ecosystem services or even use nature’s contributions to people (NCPs) [67]. Also, it would be useful to collect social preference data on a cantonal (regional) level in addition to the national level, to ensure a high quality of results on different planning scales. Moreover, scaling up this approach to a continental level, including maritime areas, would create a significant database of areas suitable for REI development, based on social and ecosystem service information. This could reshape the existing understanding of a resilient and sustainable energy transformation.

#### Meanings ascribed to energy infrastructure

The information about landscape-related social acceptance of energy infrastructure is based on people’s evaluations about the suitability of landscape(s) and REI components [54]. This so-called landscape-technology fit (LTF) is mostly predicted by meanings people ascribe to landscapes and REI. However, most of these highly relevant meanings are rooted on concepts of nature, wilderness, and landscape perception developed in the last millennium and require updating and further development to adequately reflect today’s society. Therefore, it is crucial to continually improve and refine the measurement concepts and scales to meet this challenge.

#### Temporal sensitivity of survey data

The incorporation of social preferences is shown as an interesting alternative to traditional techno-economic approaches and an urgently needed additional point of view in the decision-making process. However these data are only a snapshot, given the temporal variations in social preference data suggested by e.g. Dlamini et al. [68]. Consequently, and despite the potentially high costs of longitudinal data collection and analysis, the authors of this study emphasize that social preferences should be monitored regularly if this option is to be sustained.

At the time of this study, the Swiss population has hardly had a chance to experience real live open-space gm-PV. Most assessments are based on virtual reality, meaning that evaluated preferences are merely reflect people’s responses to experiences reported in e.g. media broadcasts and social media channels. Therefore, it would be a great benefit to the quality of the social data to monitor people’s landscape preferences during the installation phase of future gm-PV infrastructure. However, given that this study is the first to incorporate landscape preferences alongside ecosystem services for REI siting at all, future research is encouraged to apply the approach used in this study to achieve a broader perspective.

## Conclusions

The overarching insight from this study is the critical examination of the prevailing “roof first” strategy in PV development, a cornerstone of the Swiss Energy Strategy 2050 and a common approach in numerous global contexts. Contrary to its intended purpose, this strategy doesn’t effectively curtail the expansive land use for new REI development. Our analyses distinctly highlight that scenarios excluding ground-mounted PV (gm-PV) such as NRG, ESS, and SOC are notably less spatially efficient compared to those incorporating gm-PV, namely NRG-OS, ESS-OS, and SOC-OS.

The reluctance to tap into gm-PV, a significant energy resource, primarily stems from the intent to safeguard landscapes. The underlying assumption is that a reduced landscape footprint for energy transition would lead to diminished local environmental and societal impacts. However, present findings challenge this notion, emphasizing that the efficiency of a strategy isn’t solely determined by the number of Planning Units (PUs) but critically hinges on the associated costs linked to these PUs. A comparative analysis of the two social trade-off strategies, SOC-OS and SOC, reveals that the latter, despite being less spatially efficient, incurs marginally elevated ecosystem service costs and marginally reduced social costs. The differences become clearer when strategies with different focuses are also compared with each other.

Drawing from the methodological advancements and contextual discussions, this study furnishes policymakers with a nuanced understanding, presenting them with a triad of strategic choices:

1. **Spatial Concentration of REI:** This approach, likely to resonate with technocratic-economic stakeholders, emphasizes a limited number of sites. Intriguingly, while the social costs associated are minimal, the environmental costs are anticipated to be substantial. The resultant landscape transformations would be profound, with only select regions reaping economic benefits.
2. **Optimizing Ecosystem Service Costs:** Advocates for environmental conservation might gravitate towards this strategy due to its minimized ecological costs. However, it’s pertinent to note that it registers the highest social costs among the strategies explored in this study. Aligning with Switzerland’s regional-economic principles, this decentralized approach unfortunately lags in energy efficiency.
3. **Prioritizing Social Preferences:** This strategy fills a conspicuous gap in most energy discourses and stands as a testament to the study’s innovative spirit. Tailored for local and regional policymakers who champion widely accepted democratic solutions, it advocates for REI concentration in strategic areas like the plateau, the Alpine foothills, and certain urbanized Alpine valleys. A salient feature of this strategy is its preservation of iconic landscapes, emphasizing regions with shared cultural and aesthetic values. In essence, pristine landscapes remain untouched, while everyday landscapes bearing significant infrastructure bear the brunt.

Incorporating insights from the Marxan optimization approach, the study underscores the potential of this methodology in navigating the intricate trade-offs between renewable energy production, ecosystem services, and social preferences. The study further accentuates the dynamic nature of social preferences, emphasizing the need for continual monitoring and adaptation. As the energy landscape evolves, so should our strategies, ensuring they are both sustainable and socially resonant.

## Supporting information

S1 Table External costs of wind and ground-mounted photovoltaic (gm-PV) infrastructure on agricultural land, in forests, and in areas with various other land uses, expressed in external cost units (ECU_ess_) per ha. (DOCX)

S2 Table Matching the renewable energy installation (REI) attributes of the choice experiment (A) to the REI attributes of a 4 × 4 km square planning unit (B). (DOCX)

## References

[1] Suman A. Role of renewable energy technologies in climate change adaptation and mitigation: A brief review from Nepal. Renewable and Sustainable Energy Reviews. 2021;151: 111524. doi: 10.1016/j.rser.2021.111524

[2] Pani A, Shirkole SS, Mujumdar AS. Importance of renewable energy in the fight against global climate change. Drying Technology. 2022;40: 2581–2582. doi: 10.1080/07373937.2022.2119324

[3] Maisanam AKS, Biswas A, Sharma KK. Integrated socio-environmental and techno-economic factors for designing and sizing of a sustainable hybrid renewable energy system. Energy Conversion and Management. 2021;247: 114709. doi: 10.1016/j.enconman.2021.114709

[4] Nik VM, Perera ATD. The Importance of Developing Climate-Resilient Pathways for Energy Transition and Climate Change Adaptation. One Earth. 2020;3: 423–424. doi: 10.1016/j.oneear.2020.09.013

[5] Hastik R, Basso S, Geitner C, Haida C, Poljanec A, Portaccio A, et al. Renewable energies and ecosystem service impacts. Renewable and Sustainable Energy Reviews. 2015;48: 608–623. doi: 10.1016/j.rser.2015.04.004

[6] Daily GC. Nature’s Services: Societal Dependence on Natural Ecosystems (1997). In: Robin L, Sörlin S, Warde P, editors. The Future of Nature. Yale University Press; 2017. pp. 454–464.

[7] Kienast F, Huber N, Hergert R, Bolliger J, Morán LS, Hersperger AM. Conflicts between decentralized renewable electricity production and landscape services–A spatially-explicit quantitative assessment for Switzerland. RSER. 2017;67: 397–407. doi: 10.1016/j.rser.2016.09.045

[8] Huber N, Hergert R, Price B, Zäch C, Hersperger AM, Pütz M, et al. Renewable energy sources: conflicts and opportunities in a changing landscape. Reg Environ Change. 2017;17: 1241–1255. doi: 10.1007/s10113-016-1098-9

[9] Wiehe J, Thiele J, Walter A, Hashemifarzad A, Hingst J, Haaren C. Nothing to regret: Reconciling renewable energies with human wellbeing and nature in the German Energy Transition. Int J Energy Res. 2021;45: 745–758. doi: 10.1002/er.5870

[10] Egli T, Bolliger J, Kienast F. Evaluating ecosystem service trade-offs with wind electricity production in Switzerland. RSER. 2017;67: 863–875. doi: 10.1016/j.rser.2016.09.074

[11] Göke C, Dahl K, Mohn C. Maritime Spatial Planning supported by systematic site selection: Applying Marxan for offshore wind power in the western Baltic Sea. Hewitt J, editor. PLoS ONE. 2018;13: e0194362. doi: 10.1371/journal.pone.0194362 29543878 PMC5854382

[12] Lehmann P, Ammermann K, Gawel E, Geiger C, Hauck J, Heilmann J, et al. Managing spatial sustainability trade-offs: The case of wind power. Ecological Economics. 2021;185: 107029. doi: 10.1016/j.ecolecon.2021.107029

[13] Wang N, Verzijlbergh RA, Heijnen PW, Herder PM. A spatially explicit planning approach for power systems with a high share of renewable energy sources. Applied Energy. 2020;260: 114233. doi: 10.1016/j.apenergy.2019.114233

[14] Batel S. Research on the social acceptance of renewable energy technologies: Past, present and future. Energy Research & Social Science. 2020;68: 101544. doi: 10.1016/j.erss.2020.101544

[15] Baur D, Emmerich P, Baumann MJ, Weil M. Assessing the social acceptance of key technologies for the German energy transition. Energ Sustain Soc. 2022;12: 4. doi: 10.1186/s13705-021-00329-x

[16] Fouad M. Mastering the risky business of public-private partnerships in infrastructure. Washington DC: International Monetary Fund; 2021.

[17] Federal Office of Energy. Energiestrategie 2050 nach der Volksabstimmung vom 21.Mai 2017. Ittingen; 2017 Aug. http://www.bfe.admin.ch/php/modules/publikationen/stream.php?extlang=de&name=de_972399846.pdf

[18] Mearns E, Sornette D. Are 2050 energy transition plans viable? A detailed analysis of projected Swiss electricity supply and demand in 2050. Energy Policy. 2023;175: 113347. doi: 10.1016/j.enpol.2022.113347

[19] Kahl A, Dujardin J, Lehning M. The bright side of PV production in snow-covered mountains. Proc Natl Acad Sci USA. 2019;116: 1162–1167. doi: 10.1073/pnas.1720808116 30617063 PMC6347694

[20] Federal Office of Topography swisstopo. Geography—Facts and Figures. About Switzerland. Bern/CH; 2023. https://www.eda.admin.ch/aboutswitzerland/en/home/umwelt/geografie/geografie—fakten-und-zahlen.html

[21] Wachter D, Maissen T, Egli E, Diem A. Encyclopædia Britannica. Switzerland. 2020. https://www.britannica.com/place/Switzerland

[22] Federal Statistical Office. Raumgliederungen der Schweiz. Gemeindetypologie und Stadt/Land-Typologie 2012. Switzerland; 2017. https://www.bfs.admin.ch/bfs/de/home/aktuell/neue-veroeffentlichungen.assetdetail.2543323.html

[23] Federal Office of Topography swisstopo. Digital height model of Switzerland (DHM25/200m). Wabern, Switzerland: swisstopo; 2004. https://www.swisstopo.admin.ch/en/geodata/height/dhm25200.html

[24] Federal Office of Topography swisstopo. Swiss Map Vector 500. 2023. https://www.swisstopo.admin.ch/en/geodata/maps/smv/smv500.html

[25] Nematollahi S, Fakheran S, Jafari A, Pourmanafi S, Kienast F. Applying a systematic conservation planning tool and ecological risk index for spatial prioritization and optimization of protected area networks in Iran. Journal for Nature Conservation. 2022;66: 126144. doi: 10.1016/j.jnc.2022.126144

[26] Watts ME, Ball IR, Stewart RS, Klein CJ, Wilson K, Steinback C, et al. Marxan with Zones: Software for optimal conservation based land- and sea-use zoning. Environmental Modelling & Software. 2009;24: 1513–1521. doi: 10.1016/j.envsoft.2009.06.005

[27] Harris LR, Watts ME, Nel R, Schoeman DS, Possingham HP. Using multivariate statistics to explore trade-offs among spatial planning scenarios. Armsworth P, editor. J Appl Ecol. 2014;51: 1504–1514. doi: 10.1111/1365-2664.12345

[28] Marxan. Marxan conservation solutions. 2020. https://marxansolutions.org/

[29] Ardron JA, Possingham HP, Klein CJ, editors. Marxan Good Practices Handbook. Victoria, BC, Canada: Pacific Marine Analysis and Research Association (PacMARA); 2013. https://pacmara.org/

[30] Götz L. Applying a Systematic Conservation-Planning Tool with Real Data of Canton Aargau. Swiss Federal Research Institute WSL. 2014. https://ethz.ch/content/dam/ethz/special-interest/usys/ites/ites-dam/Education/Portal%20Forest%20and%20Landscape/Documents/MA_Abstracts_2015/MA_GL.pdf

[31] Jaeger JAG, Bertiller R, Schwick C, Cavens D, Kienast F. Urban permeation of landscapes and sprawl per capita: New measures of urban sprawl. Ecological Indicators. 2010;10: 427–441. doi: 10.1016/j.ecolind.2009.07.010

[32] Li M, McGrath H, Stefanakis E. Multi-resolution topographic analysis in hexagonal Discrete Global Grid Systems. International Journal of Applied Earth Observation and Geoinformation. 2022;113: 102985. doi: 10.1016/j.jag.2022.102985

[33] Burdziej J. Using hexagonal grids and network analysis for spatial accessibility assessment in urban environments–a case study of public amenities in Toruń. Miscellanea Geographica. 2019;23: 99–110. doi: 10.2478/mgrsd-2018-0037

[34] Angelis-Dimakis A, Biberacher M, Dominguez J, Fiorese G, Gadocha S, Gnansounou E, et al. Methods and tools to evaluate the availability of renewable energy sources. Renewable and Sustainable Energy Reviews. 2011;15: 1182–1200. doi: 10.1016/j.rser.2010.09.049

[35] Bundesamt für Energie BFE. Windatlas Schweiz. 2019. https://opendata.swiss/de/dataset/windatlas-schweiz-jahresmittel-der-modellierten-windgeschwindigkeit-und-windrichtung-in-50-m-ho

[36] Hergert R. Erneuerbare Energien aus der Landschaft Schweiz. Potentialberechnung unter Berücksichtigung verschiedener landschaftlicher Ausschlusskriterien und künftig nutzbarer Flächen. 2013. https://www.wsl.ch/fileadmin/user_upload/WSL/Ueber_die_WSL/Forschungsprogramme_Initiativen/Zentrum_Landschaft/Masterarbeiten_Zentrum_Landaschaft/Hergert_Rico_Masterarbeit.pdf

[37] Kienast F, Hersperger AM, Hergert R, Segura Moran L. Landschaftskonflikte durch erneuerbare Energien. Swiss Federal Institute for Forest, Snow and Landscape Research WSL, editor. Berichte WSL. 2014;21: 69–74.

[38] Klauser D. Solarpotentialanalyse für Sonnendach.ch. Bern/CH; 2016 Feb p. 97. Report No.: SI/300186-01. https://pubdb.bfe.admin.ch/de/publication/download/8196

[39] BFS Geostat. Arealstatistik 2004/2009. 2004.

[40] Lütkehus I, Salecker C, Adlunger K. Potenzial der Windenergier an Land: Studie zur Ermittlung des Bundesweiten Flächen-und Leistungspotenzials der Windenergienutzung an Land. Dessau-Roßlau; 2013 Jun p. 51. Available: https://www.umweltbundesamt.de/sites/default/files/medien/378/publikationen/potenzial_der_windenergie.pdf

[41] Jongbloed RH, van der Wal JT, Lindeboom HJ. Identifying space for offshore wind energy in the North Sea. Consequences of scenario calculations for interactions with other marine uses. Energy Policy. 2014;68: 320–333. doi: 10.1016/j.enpol.2014.01.042

[42] European Environment Agency EEA. CICES—common classification of ecosystem services. 2017. https://cices.eu/

[43] Trommsdorff M, Dhal IS, Özdemir ÖE, Ketzer D, Weinberger N, Rösch C. Agrivoltaics: solar power generation and food production. Solar Energy Advancements in Agriculture and Food Production Systems. Elsevier; 2022. pp. 159–210. doi: 10.1016/B978-0-323-89866-9.00012-2

[44] Waghmare RM, Jilte R, Joshi S. Performance analysis of Agrophotovoltaic systems with Solanum lycopersicum crops. Materials Today: Proceedings. 2023;72: 1284–1289. doi: 10.1016/j.matpr.2022.09.300

[45] Horch P, Schmid H, Guélat J, Liechti F. Konfliktpotenzialkarte Windenergie–Vögel Schweiz: Teilbereich Brutvögel, Gastvögel und Vogelschutzgebiete gemäss WZVV. Erläuterungsbericht. Aktualisierung 2013. Schweizerische Vogelwarte, Sempbach; 2013. https://www.vogelwarte.ch/assets/files/projekte/konflikte/konfliktpotenzialkarte/Bericht_KonfliktpotenzialkarteCH_BVGVGE_2013.pdf

[46] Liechti F, Guélat J, Komenda-Zehnder S. Modelling the spatial concentrations of bird migration to assess conflicts with wind turbines. Biological Conservation. 2013;162: 24–32. doi: 10.1016/j.biocon.2013.03.018

[47] Federal Commission for the Protection of Nature and Cultural Heritage (FCNC). Landscapes and natural monuments. Bern/CH; 2020. https://www.enhk.admin.ch/en/topics/landscapes-and-natural-monuments

[48] Stiftung SchweizMobil. Schweiz Mobil. Varoious outdoor routes. 2023. https://map.schweizmobil.ch/?lang=de&photos=yes&logo=yes&detours=yes&season=summer&bgLayer=pk&resolution=250&E=2631750&N=1189000

[49] Federal Statistical Office. Hotel accommodation: arrivals and overnight stays of open establishments by year, month, canton and visitors’ country of residence. 2023 Apr. Report No.: px-x-1003020000_102. https://www.bfs.admin.ch/bfs/en/home/statistics/tourism/tourist-accommodation.assetdetail.24805214.html

[50] World Heritage Experience Switzerland—WHES. World Heritage Sites. 2023. https://ourheritage.ch/map

[51] Swiss Federal Office of Topography (swisstopo). The Topographic Landscape Model—swissTLM3D 2.1. Wabern, Switzerland; 2023. https://www.swisstopo.admin.ch/en/knowledge-facts/topographic-landscape-model.html

[52] Federal Office of Culture. Federal Inventory of Heritage Sites of national importance ISOS and protection of heritage sites. Bern; 2023. https://www.bak.admin.ch/bak/en/home/baukultur/isos-und-ortsbildschutz.html

[53] Salak B, Kienast F, Olschewski R, Spielhofer R, Wissen Hayek U, Grêt-Regamey A, et al. Impact on the perceived landscape quality through renewable energy infrastructure. A discrete choice experiment in the context of the Swiss energy transition. RENE. 2022;193: 299–308. doi: 10.1016/j.renene.2022.04.154

[54] Salak B, Lindberg K, Kienast F, Hunziker M. How landscape-technology fit affects public evaluations of renewable energy infrastructure scenarios. A hybrid choice model. Renewable and Sustainable Energy Reviews. 2021;143: 110896. doi: 10.1016/j.rser.2021.110896

[55] Salak B, Lindberg K, Kienast F, Hunziker M. Hybrid choice model dataset of a representative Swiss online panel survey on peoples’ preferences related to mixed renewable energy scenarios in landscapes and the effect of landscape-technology fit. Data in Brief. 2021;36: 107025. doi: 10.1016/j.dib.2021.107025 34026963 PMC8131564

[56] Spielhofer R, Thrash T, Hayek UW, Grêt-Regamey A, Salak B, Grübel J, et al. Physiological and behavioral reactions to renewable energy systems in various landscape types. Renewable and Sustainable Energy Reviews. 2021;135: 110410. doi: 10.1016/j.rser.2020.110410

[57] Allenby GM, Arora N, Ginter JL. Incorporating Prior Knowledge into the Analysis of Conjoint Studies. Journal of Marketing Research. 1995;32: 152. doi: 10.2307/3152044

[58] Lenk PJ, DeSarbo WS, Green PE, Young MR. Hierarchical Bayes Conjoint Analysis: Recovery of Partworth Heterogeneity from Reduced Experimental Designs. Marketing Science. 1996;15: 173–191. doi: 10.1287/mksc.15.2.173

[59] Orme BK. Getting started with conjoint analysis: strategies for product design and pricing research. 3. ed. Glendale, Calif: Research Publ; 2014.

[60] Janßen H, Göke C, Luttmann A. Knowledge integration in Marine Spatial Planning: A practitioners’ view on decision support tools with special focus on Marxan. Ocean & Coastal Management. 2019;168: 130–138. doi: 10.1016/j.ocecoaman.2018.11.006

[61] Ashbolt SC, Maheepala S, Perera BJC. Interpreting a Pareto set of operating options for water grids: a framework and case study. Hydrological Sciences Journal. 2017;62: 2631–2654. doi: 10.1080/02626667.2017.1398826

[62] Seppelt R, Lautenbach S, Volk M. Identifying trade-offs between ecosystem services, land use, and biodiversity: a plea for combining scenario analysis and optimization on different spatial scales. Current Opinion in Environmental Sustainability. 2013;5: 458–463. doi: 10.1016/j.cosust.2013.05.002

[63] Spielhofer R, Schwaab J, Grêt-Regamey A. How spatial policies can leverage energy transitions − Finding Pareto-optimal solutions for wind turbine locations with evolutionary multi-objective optimization. Environmental Science & Policy. 2023;142: 220–232. doi: 10.1016/j.envsci.2023.02.016

[64] Kati V, Kassara C, Vrontisi Z, Moustakas A. The biodiversity-wind energy-land use nexus in a global biodiversity hotspot. Science of The Total Environment. 2021;768: 144471. doi: 10.1016/j.scitotenv.2020.144471 33454485

[65] Liu L, Stevens RJAM. Effects of Two-Dimensional Steep Hills on the Performance of Wind Turbines and Wind Farms. Boundary-Layer Meteorol. 2020;176: 251–269. doi: 10.1007/s10546-020-00522-z

[66] Brudermann T, Zaman R, Posch A. Not in my hiking trail? Acceptance of wind farms in the Austrian Alps. Clean Techn Environ Policy. 2019;21: 1603–1616. doi: 10.1007/s10098-019-01734-9

[67] Brück M, Abson DJ, Fischer J, Schultner J. Broadening the scope of ecosystem services research: Disaggregation as a powerful concept for sustainable natural resource management. Ecosystem Services. 2022;53: 101399. doi: 10.1016/j.ecoser.2021.101399

[68] Dlamini S, Tesfamichael SG, Breetzke GD, Mokhele T. Spatio-temporal patterns and changes in environmental attitudes and place attachment in Gauteng, South Africa. Geo-spatial Information Science. 2021;24: 666–677. doi: 10.1080/10095020.2021.1976599

