# Supplementary material for: Shifting from techno-economic to socio-ecological priorities: Incorporating landscape preferences and ecosystem services into the siting of renewable energy infrastructure
Source: PLoS One. 2024 Apr 10;19(4):e0298430. doi: 10.1371/journal.pone.0298430 (PMC11006175; doi:10.1371/journal.pone.0298430)
Supplement: S1 Table — (DOCX) [file pone.0298430.s001.docx]

**S1 Table.** **External costs of wind and ground-mounted photovoltaic (gm-PV) infrastructure on agricultural land, in forests, and in areas with various other land uses, expressed in external cost units (ECU_ess_) per ha.** A scale of 0 (lowest) to 4 (highest) was used. Detailed arguments to using these semiquantitative scales see text.

|  |  | Wind | gm-PV | |
| --- | --- | --- | --- | --- |
| Number | Land use | Externalities concerning food and biomass production (ECU_ess_ per 1 ha pixel) | Externalities concerning food and biomass production (ECU_ess_ per 1 ha pixel) | Externalities concerning lifecycle maintenance and habitat and gene pool protection (ECU_ess_ per 1 ha pixel) |
| 1 | Industrial and commercial buildings | masked out | masked out | masked out |
| 2 | Surroundings of industrial and commercial buildings | masked out | masked out | masked out |
| 3 | One- and two-family houses | masked out | masked out | masked out |
| 4 | Surroundings of one- and two-family houses | masked out | masked out | masked out |
| 5 | Terraced houses | masked out | masked out | masked out |
| 6 | Surroundings of terraced houses | masked out | masked out | masked out |
| 7 | Blocks of flats | masked out | masked out | masked out |
| 8 | Surroundings of blocks of flats | masked out | masked out | masked out |
| 9 | Public buildings | masked out | masked out | masked out |
| 10 | Surroundings of public buildings | masked out | masked out | masked out |
| 11 | Agricultural buildings | masked out | masked out | masked out |
| 12 | Surroundings of agricultural buildings | masked out | masked out | masked out |
| 13 | Unspecified buildings | masked out | masked out | masked out |
| 14 | Surroundings of unspecified buildings | masked out | masked out | masked out |
| 15 | Motorways | masked out | masked out | masked out |
| 16 | Green motorway environs | masked out | 0; 1^a^ | 2; 4^a^ |
| 17 | Roads and paths | masked out | masked out | masked out |
| 18 | Green road environs | masked out | masked out | masked out |
| 19 | Parking areas | masked out | 0 | 0 |
| 20 | Sealed railway areas | masked out | masked out | masked out |
| 21 | Green railway environs | masked out | 0; 1^a^ | 2; 4^a^ |
| 22 | Airports | masked out | masked out | masked out |
| 23 | Airfields | masked out | masked out | masked out |
| 24 | Energy supply plants | masked out | masked out | masked out |
| 25 | Waste water treatment plants | masked out | masked out | masked out |
| 26 | Other supply and waste treatment plants | masked out | masked out | masked out |
| 27 | Dumps | masked out | masked out | masked out |
| 28 | Quarries | masked out | masked out | masked out |
| 29 | Construction sites | masked out | masked out | masked out |
| 30 | Unexploited urban areas | masked out | masked out | masked out |
| 31 | Public parks | masked out | masked out | masked out |
| 32 | Sports facilities | masked out | masked out | masked out |
| 33 | Golf courses | masked out | masked out | masked out |
| 34 | Camping areas | masked out | masked out | masked out |
| 35 | Garden allotments | masked out | masked out | masked out |
| 36 | Cemeteries | masked out | masked out | masked out |
| 37 | Intensive orchards | masked out | masked out | masked out |
| 38 | Field fruit trees | masked out | masked out | masked out |
| 39 | Vineyards | masked out | masked out | masked out |
| 40 | Horticulture | masked out | 3 | 0 |
| 41 | Arable land | 1 | 2; 1^a^ | 0; 4^a^ |
| 42 | Meadows | 1 | 2; 1^a^ | 0; 4^a^ |
| 43 | Farm pastures | 3 | 1; 1^a^ | 0; 4^a^ |
| 44 | Brush meadows and farm pastures | 1 | 0; 1^a^ | 3; 4^a^ |
| 45 | Alpine meadows | 1 | 2; 1^a^ | 0; 4^a^ |
| 46 | Favorable alpine pastures | 1 | 2; 1^a^ | 0; 4^a^ |
| 47 | Brush alpine pastures | 1 | masked out | masked out |
| 48 | Rocky alpine pastures | 1 | masked out | masked out |
| 49 | Sheep pastures | 1 | masked out | masked out |
| 50 | Closed forest | 1 | masked out | masked out |
| 51 | Forest strips | 1 | masked out | masked out |
| 52 | Afforestations | 1 | masked out | masked out |
| 53 | Felling areas | 1 | masked out | masked out |
| 54 | Damaged forest areas | 1 | masked out | masked out |
| 55 | Open forest (in agricultural areas) | 1 | 0; 1^a^ | 2; 4^a^ |
| 56 | Open forest (in unproductive areas) | 1 | 0; 1^a^ | 2; 4^a^ |
| 57 | Brush forest | 1 | masked out | masked out |
| 58 | Groves | 1 | masked out | masked out |
| 59 | Clusters of trees (in agricultural areas) | 1 | masked out | masked out |
| 60 | Clusters of trees (in unproductive areas) | 1 | masked out | masked out |
| 61 | Lakes | masked out | masked out | masked out |
| 62 | Rivers | masked out | masked out | masked out |
| 63 | Flood protection structures | masked out | masked out | masked out |
| 64 | Scrub vegetation | 0 | 0; 1^a^ | 3; 4^a^ |
| 65 | Unproductive grass and shrubs | 0 | masked out | masked out |
| 66 | Avalanche and rockfall barriers | masked out | masked out | masked out |
| 67 | Wetlands | masked out | masked out | masked out |
| 68 | Alpine sports facilities | masked out | masked out | masked out |
| 69 | Rocks | 0 | masked out | masked out |
| 70 | Scree | masked out | masked out | masked out |
| 71 | Landscape interventions | masked out | masked out | masked out |
| 72 | Glaciers | masked out | masked out | masked out |
| ^a^If a pixel belongs to the inventory of dry grasslands of national importance, the higher value applies. | | | | |
