# Supplementary material for: Shifting from techno-economic to socio-ecological priorities: Incorporating landscape preferences and ecosystem services into the siting of renewable energy infrastructure
Source: PLoS One. 2024 Apr 10;19(4):e0298430. doi: 10.1371/journal.pone.0298430 (PMC11006175; doi:10.1371/journal.pone.0298430)
Supplement: S2 Table — (DOCX) [file pone.0298430.s002.docx]

**S2 Table.** **Matching the renewable energy installation (REI) attributes of the choice experiment (A) to the REI attributes of a 4 × 4 km square planning unit (B)**. gm-PV = ground-mounted photovoltaic, rm-PV = roof-mounted photovoltaic.

| **A** | | | |  | **B** | | |
| --- | --- | --- | --- | --- | --- | --- | --- |
| **Spatially non-explicit discrete choice experiment attributes** | | | **Scenario number** |  | **Spatially explicit attributes for 1 planning unit (4 × 4 km) with max. 99 hexagons, each with max. 1 wind turbine** | | |
| *Attribute landscape* | *Attribute wind energy* | *Attribute PV* |  |  | *Number of hexagons with … rm-PV* | *Number of hexagons with ... gm-PV* | *Number of hexagons with a wind turbine* |
| **1 JURA**  **2 PLAT_URB**  **3 PLAT_AGRI**  **4 ALP_URB**  **5 ALP_TOUR**  **6 PRE_ALPS**  **7 ALP_INF**  **8 ALP** | NO: no wind turbines | NO: no PVs | 1 |  | 0 | 0 | 0 |
|  |  | MIN: small to medium number of PVs | 2 |  | 4–99 (max.) |  |  |
|  |  |  |  |  | 0–49 | 1–2 |  |
|  |  | MED medium to large PVs | 3 |  | 50–99 (max.) |  |  |
|  |  |  |  |  | 0–49 | 3–4 |  |
|  |  | MAX: high PV | 4 | <-> | 50–99 (max.) |  |  |
|  | MIN small number of wind turbines | NO | 5 |  | 0–3 | 0 | 2–3 |
|  |  | MIN | 6 |  | 4–99 (max.) |  |  |
|  |  |  |  |  | 0–49 | 1–2 |  |
|  |  | MED | 7 |  | 50–99 (max.) |  |  |
|  |  |  |  |  | 0–49 | 3–4 |  |
|  |  | MAX | 8 |  | 50–99 (max.) |  |  |
|  | MED Average number of wind turbines | NO | 9 |  | 0–3 | 0 | 4–6 |
|  |  | MIN | 10 |  | 4–99 (max.) |  |  |
|  |  |  |  |  | 0–49 | 1–2 |  |
|  |  | MED | 11 |  | 50–99 (max.) |  |  |
|  |  |  |  |  | 0–49 | 3–4 |  |
|  |  | MAX | 12 |  | 50–99 (max.) |  |  |
|  | MAX large number of wind turbines | NO | 13 |  | 0–3 | 0 | >7 |
|  |  | MIN | 14 |  | 4–99 (max.) |  |  |
|  |  |  |  |  | 0–49 | 1–2 |  |
|  |  | MED | 15 |  | 0–3 |  |  |
|  |  |  |  |  | 4–99 (max.) | 3–4 |  |
|  |  | MAX | 16 |  | 0–49 |  |  |
